# Global research trends in microbiome-gut-brain axis during 2009–2018: a bibliometric and visualized study

**DOI:** 10.1186/s12876-019-1076-z

**Published:** 2019-08-30

**Authors:** Sa’ed H. Zyoud, Simon Smale, W. Stephen Waring, Waleed M. Sweileh, Samah W. Al-Jabi

**Affiliations:** 1 Poison Control and Drug Information Center (PCDIC), College of Medicine and Health Sciences, An-Najah National University, Nablus, 44839 Palestine; 2 Department of Clinical and Community Pharmacy, College of Medicine and Health Sciences, An-Najah National University, Nablus, 44839 Palestine; 3 Clinical Research Centre, An-Najah National University Hospital, Nablus, 44839 Palestine; 4 Department of Gastroenterology, York Hospital, York Teaching Hospital NHS Foundation Trust, Wigginton Road, York, YO31 8HE UK; 5 Acute Medical Unit, York Teaching Hospitals NHS Foundation Trust, Wigginton Road, York, YO31 8HE UK; 6 Department of Pharmacology and Toxicology, College of Medicine and Health Sciences, An-Najah National University, Nablus, 44839 Palestine

**Keywords:** Gut microbiota, Gut microbiome-brain axis, Microbiome, Bibliometric, Scopus

## Abstract

**Background:**

The pathways and mechanism by which associations between the gut microbiome and the brain, termed the microbiome-gut-brain axis (MGBA), are manifest but remain to be fully elucidated. This study aims to use bibliometric analysis to estimate the global activity within this rapidly developing field and to identify particular areas of focus that are of current relevance to the MGBA during the last decade (2009–2018).

**Methods:**

The current study uses the Scopus for data collection. We used the key terms “microbiome-gut-brain axis” and its synonyms because we are concerned with MGBA per se as a new concept in research rather than related topics. A VOSviewer version 1.6.11 was used to visualize collaboration pattern between countries and authors, and evolving research topics by analysis of the term co-occurrence in the title and abstract of publications.

**Results:**

Between 2009 and 2018, there were 51,504 published documents related to the microbiome, including 1713 articles related to the MGBA: 829 (48.4%) original articles, 658(38.4%) reviews, and 226 (13.2%) other articles such as notes, editorials or letters. The USA took the first place with 385 appearances, followed by Ireland (*n* = 161), China (*n* = 155), and Canada (*n* = 144).The overall citation h-index was 106, and the countries with the highest h-index values were the USA (69), Ireland (58), and Canada (43). The cluster analysis demonstrated that the dominant fields of the MGBA include four clusters with four research directions: “modeling MGBA in animal systems”, “interplay between the gut microbiota and the immune system”, “irritable bowel syndrome related to gut microbiota”, and “neurodegenerative diseases related to gut microbiota”.

**Conclusions:**

This study demonstrates that the research on the MGBA has been becoming progressively more extensive at global level over the past 10 years. Overall, our study found that a large amount of work on MGBA focused on immunomodulation, irritable bowel syndrome, and neurodevelopmental disorders. Despite considerable progress illustrating the communication between the gut microbiome and the brain over the past 10 years, many issues remain about their relevance for therapeutic intervention of many diseases.

## Background

The interaction between gut and brain has been acknowledged by physicians since antiquity [1]. As far back as the sixteenth century, the association between depression and altered bowel function was recognized and in 1978 Manning and his colleagues described the “irritable bowel syndrome (IBS)” as a gastrointestinal condition which is strongly associated with psychological stress, some authors reporting 50% of sufferers have comorbid depression or anxiety [2]. The pathways and mechanism by which these associations are manifest remain to be fully elucidated. However, recent developments in genome sequencing, metabolomics, functional imaging and computational biology have increased our understanding considerably [3–6].

The rapid development of 16S ribosomal RNA and whole genome sequencing analysis has enabled us to understand the diverse nature of the microbial symbionts that inhabit our gastrointestinal tract [7–9]. Metabolomics is beginning to explain how those microbes produce a range of molecules that impact our behaviors and perceptions. The changes in our microbial diversity, manifest as changes in their metabolic output appear to alter the development of multiple facets of the enteric and central nervous systems including astrocytes, microglial cells and neurons [10, 11]. Functional imaging, functional magnetic resonance imaging and magneto encephalography, have enabled us to identify real time changes in neurological activity and correlate these with changes in behavior or perception [12–14]. Advances in computational biology are beginning to explain how these multifaceted and complex systems interact with each other [15, 16].

The microbiota interacts with the host through their effect on immune, neuro-hormonal and neural pathways. They have been shown to impact a broad range of disease, including neurodegenerative disorders, such as multiple sclerosis and Parkinson’s disease, auto-immune disease and obesity [17, 18]. The gastrointestinal microbiome has also been shown to influence behavior in mammals and man [19, 20]. Transfer of feces from depressed humans to microbiota depleted rats led the recipient rats to display behaviors analogous to depression in the human (anhedonia and anxiety like behaviors) [21, 22]. A strain of bifidobacteria has been demonstrated to increase resilience in people with anxiety [23]. These findings were not observed when healthy people consumed a strain of *Lactobacillus* [24]. Short chain fatty acids, propionate, butyrate and acetate, are important products of the microbiome and changes in the proportion and quantities of these products alter insulin resistance, ghrelin production and presumably appetite and risk of obesity and diabetes [25, 26].

Bibliometric analyses have been used in various fields to highlight the most influential countries, authors, journals, publications, and institutions [27–42]. These include research related to microbiota [43, 44]. Worldwide, there are more than 330 clinical studies recorded on clinical trials.gov with a specific focus on the microbiome. This is a growing area of importance in order to better understand the impact of specific strains on individuals, and the interaction with pre-existing microbial symbionts. Currently, there is a lack of research concerning assessment of the current status, hot spots, and future outlook on the theme of the microbiome-gut-brain axis (MGBA). This study aims to use bibliometric methods to identify particular areas of research activity in this field and to allow researchers to identify new areas for future development.

## Methods

Although a large number of databases are used for evaluation research at global level [45–47], the current study uses the Scopus database which is widely accepted among researchers for the purposes of high quality bibliometric analyses [44, 48–53]. Scopus is the world’s largest abstract and citation database of peer-reviewed research literature, and is an established resource for identifying biomedical research including MEDLINE documents, and includes a higher level of detail than PubMed including the country of origin and citations per document [47, 54].

We used the key terms “microbiome-gut-brain axis” and its synonyms because we are concerned with microbiome-gut-brain axis per se as a new concept in research rather than related topics. Data mining was conducted on July 12, 2019. The central theme in this study was research articles containing “microbiome or microbiota and brain-gut or gut-brain” to identify items based on their search in the fields title, abstract and keyword simultaneously and the time was 10 years between 2009 and 2018.

### Data analysis

VOSviewer software (www.vosviewer.com, Van Eck & Waltman version 1.6.11) was used to create a visual representation of collaborations between countries and authors using network maps [55]. Creating a term co-occurrence map in VOSviewer involved only terms that occurred in the title and abstract at least 50 times under binary counting [55]. Terms with the highest relevance score were used to create a term map for network visualization. The algorithm was designed to ensure that terms that co-occurred more frequently had larger bubbles and terms that have a high similarity are located close to each other [55].

Statistical analysis was carried out for the retrieved data by the Statistical Package for the Social Sciences (version 16.0, SPSS Inc., Chicago, IL, USA). Pearson correlation Coefficient was used to test the correlation between some variables (e.g. h-index and number of publications for each country, number of publications and years, and the number of publications related to MGBA and the number of publications related to microbiome in all fields). The analyses carried out in the current study focused largely on the frequencies and percentages of publications for types of documents, countries, journals, and institutes.

## Results

**Fig. 1:**
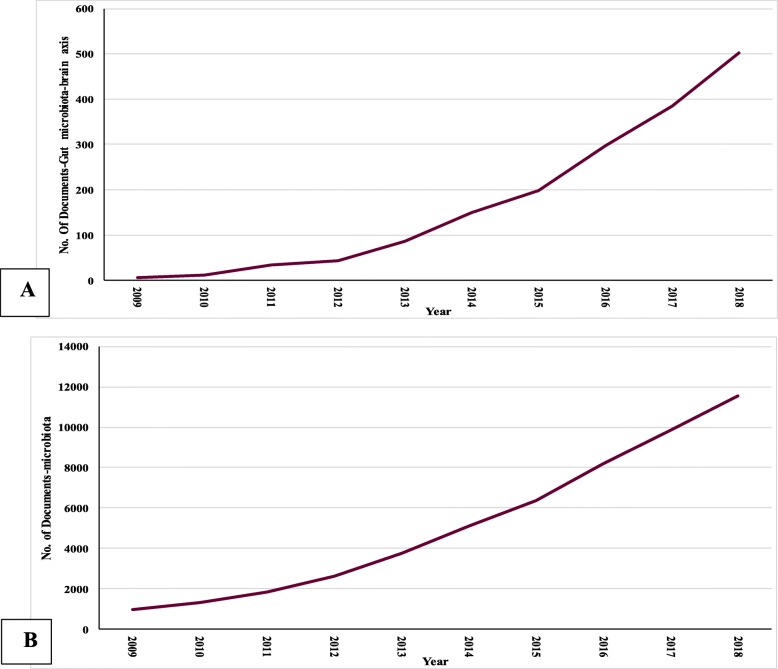
Quantitative growth process of the publications concerning microbiome-gut-brain axis (**a**) and microbiome in all fields (**b**) in the period of 10 years

Between 2009 and 2018, there were 51,504 published documents related to the microbiome, including 1713 articles related to the MGBA: 829 (48.4%) original articles, 658(38.4%) reviews, and 226 (13.2%) other articles such as notes, editorials or letters. English was the most frequently used language (*n* = 1648), followed by French (*n* = 16), and Chinese (*n* = 19), with these accounting for 98.2% of publications related to MGBA. Publications related to MGBA and the microbiome are represented in Fig. 1a and b, respectively. Time trend analyses show rising numbers of publications related to MGBA between 2009 and 2018 (*r* = 0.950; *P* value< 0.001), and a correlation between overall numbers of microbiome and MGBA publications (*r* = 0.991, *p* < 0.001) during the study period.

**Fig. 2:**
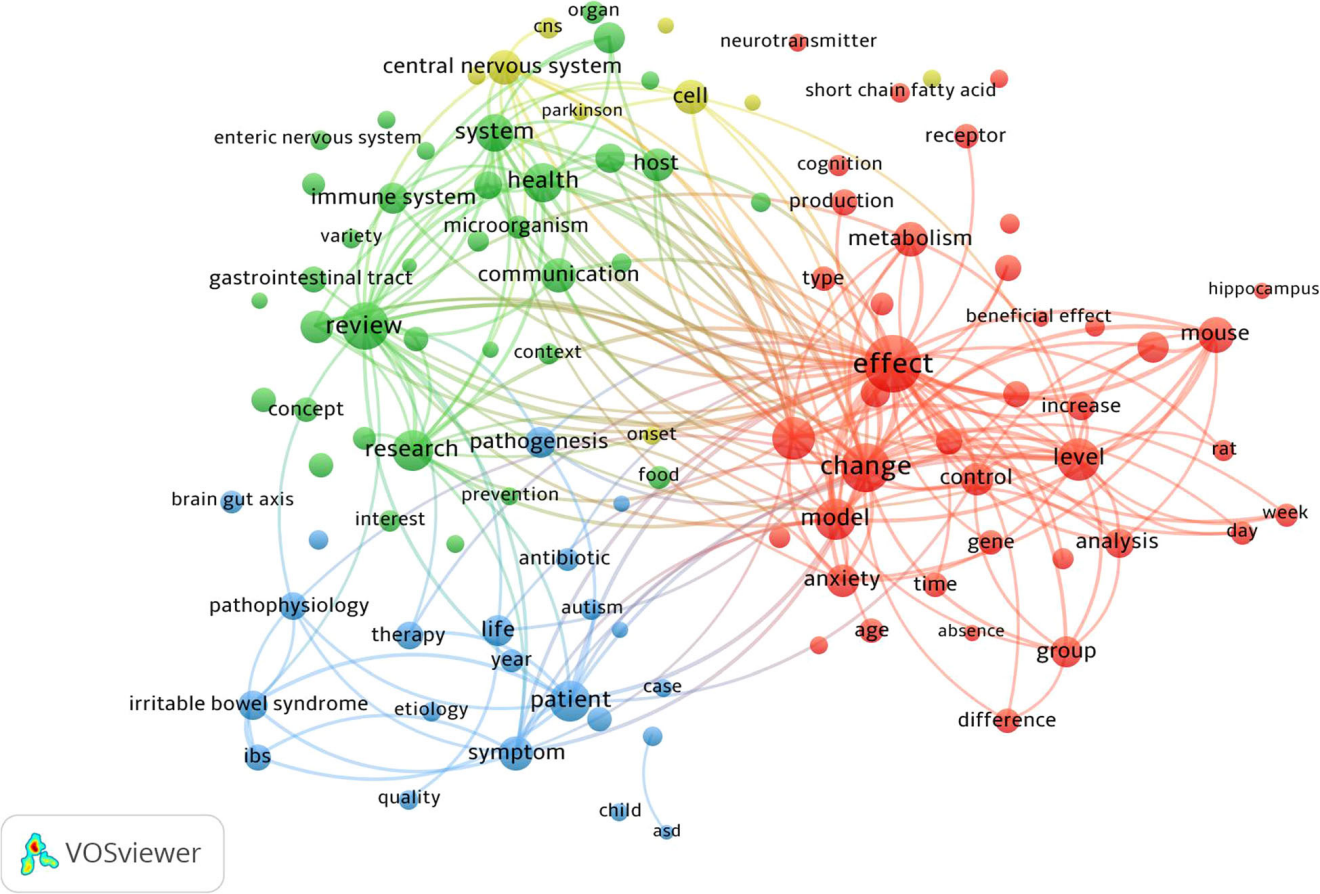
Research topics clustered by mapping of co-occurrences of terms in title/abstract for publications related to microbiome-gut-brain axis (MGBA). Of the 30,250 terms, 179 terms occurred at least 50 times. For each of the 179 terms, a relevance score was calculated and used to select the 60% most relevant terms. In Fig. 2, the size of the circles represents the occurrences of terms in title/abstract. The largest set of connected terms consists of 107 terms in four clusters. The four clusters can be broadly interpreted as “modeling MGBA in animal systems (red cluster)”, “interplay between the gut microbiota and the immune system (green cluster)”, “irritable bowel syndrome related to gut microbiota (blue cluster)”, and “neurodegenerative diseases related to gut microbiota (yellow cluster)”

The term analyses maps are presented in Fig. 2: the larger circles representing frequently occurring abstract and title terms. Colors used to differentiate between 4 main topic clusters: 1. “modeling MGBA in animal systems (red cluster)”, 2. “interplay between the gut microbiota and the immune system (green cluster)”, 3. “irritable bowel syndrome related to gut microbiota (blue cluster)”, and 4. “neurodegenerative diseases related to gut microbiota (yellow cluster)”.

**Fig. 3:**
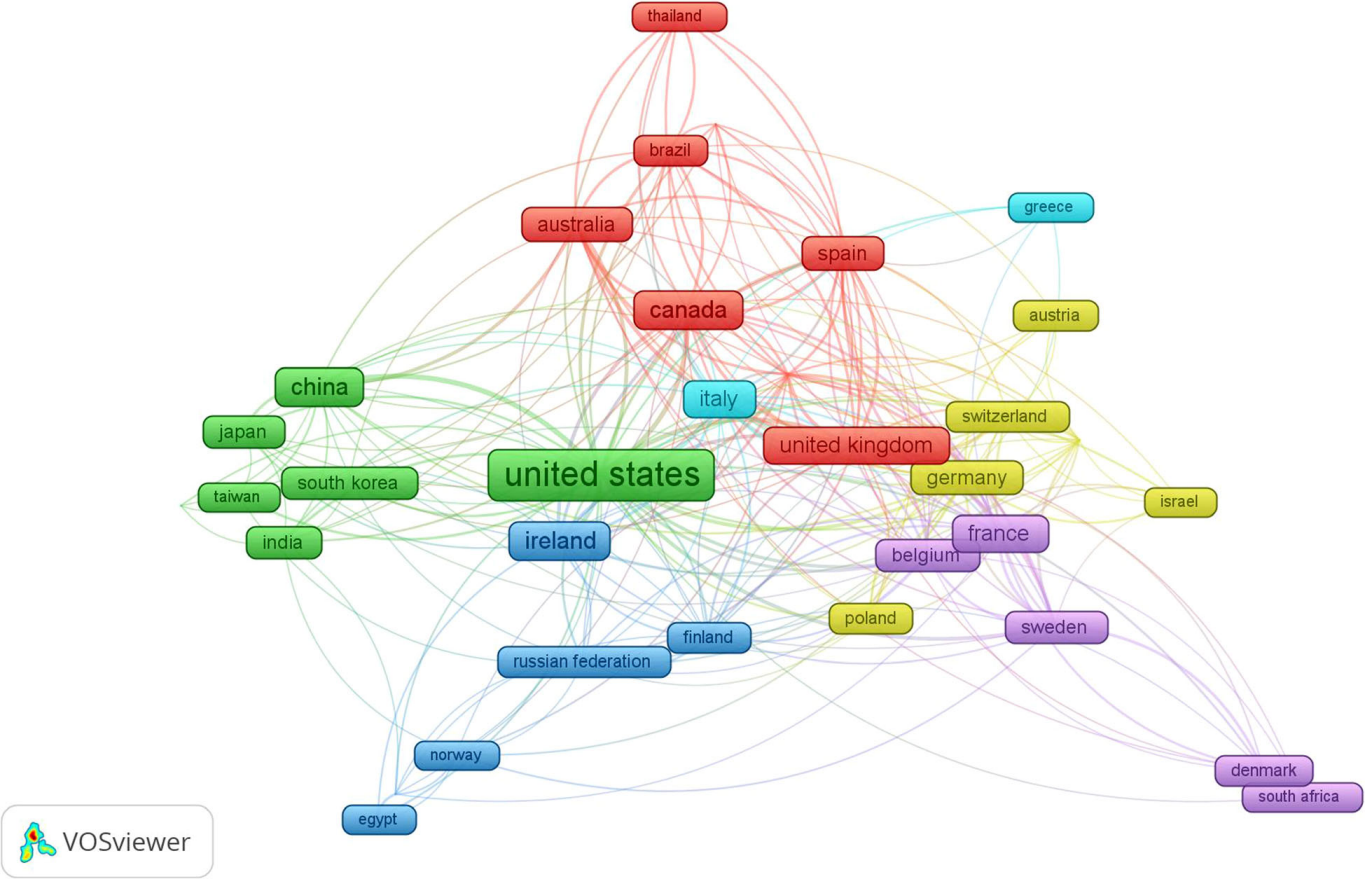
Network visualization map for country collaboration. Of the 86 countries, 35 had at least ten publications; the largest set of connected countries consists of 34 countries. The size of frame represents the number of publications of the country and the thickness of lines signifies the size of collaboration between the countries, while 6 different colors seen in this figure represent the collaboration cluster of the countries

Table 1 presents the 10 most prolific countries related to MGBA publications, with the top 4 being the USA (*n* = 385), Ireland (*n* = 161), China (*n* = 155), and Canada (*n* = 144). The overall citation h-index was 106, and the countries with the highest h-index values were the USA (69), Ireland (58), and Canada (43). There is a positive modest correlation between h-index and number of published articles (*r* = 0.817, *P*-value = 0.004). Figure 3 shows the network visualization map for country collaborations, showing 35 out of a total 86 countries that had more than ten publications; the size of frame represents the number of publications, the thickness of lines signifies the extent of collaboration between the countries.

**Table 1:** Ten leading countries in the publications concerning microbiome-gut-brain axis

SCR	Country	Number of documents (%)	*h*-index	No. of collaborated countries	No. of articles from collaboration
1st	United States	585 (34.2)	69	48	189
2nd	Ireland	161 (9.4)	58	21	58
3rd	China	155 (9.1)	28	22	56
4th	Canada	144 (8.4)	43	30	67
5th	United Kingdom	127 (7.4)	37	31	83
6th	Italy	121 (7.1)	26	28	42
7th	France	102 (6.0)	29	28	48
8th	Australia	82 (4.8)	25	19	43
9th	Germany	81 (4.7)	24	24	45
10th	Spain	65 (3.8)	21	29	34

*SCR* Standard competition ranking

**Fig. 4:**
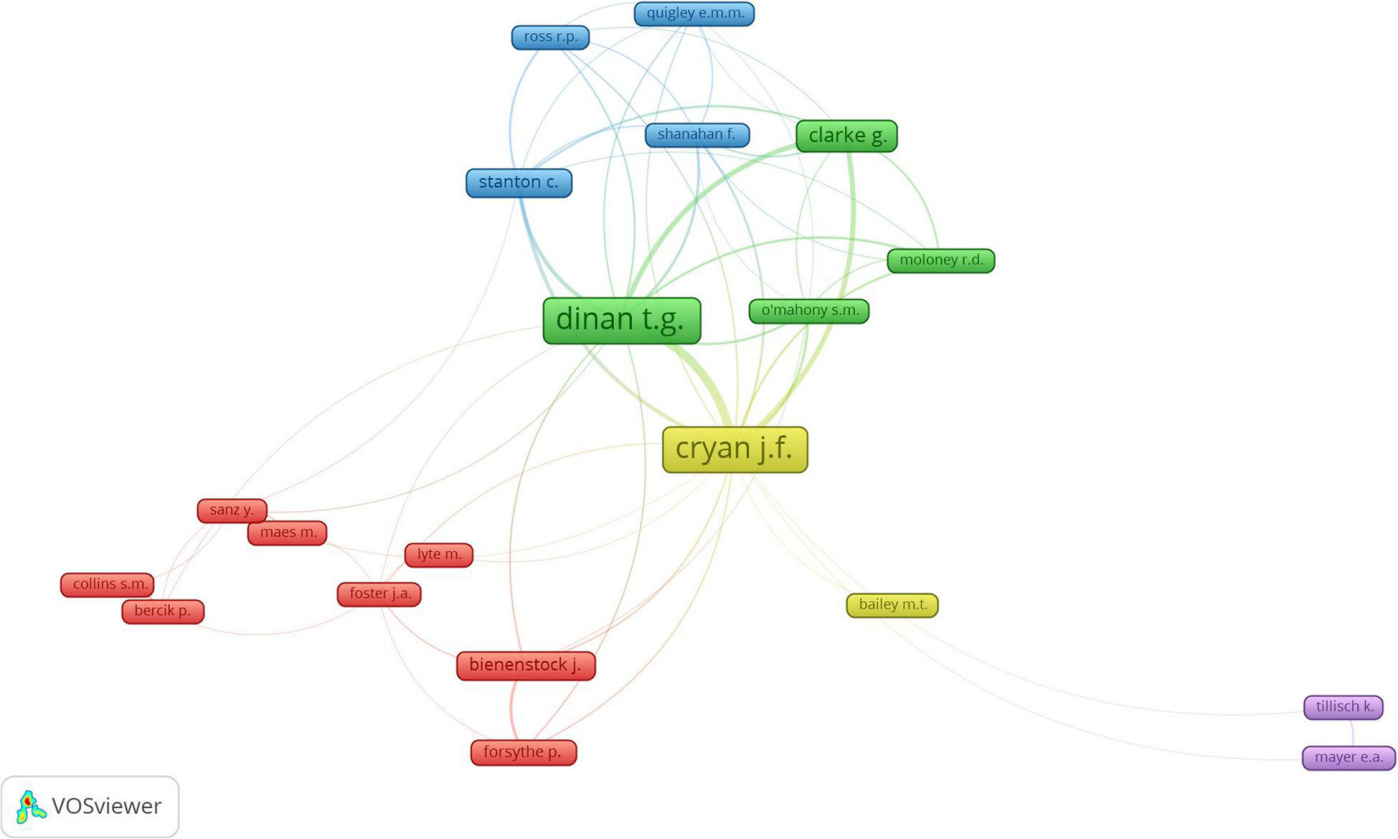
Network visualization map for author collaboration. Of the 6054 authors, 25 had at least ten publications; the largest set of connected authors consists of 20 authors. The size of frame represents the number of publications of the author and the thickness of lines signifies the size of collaboration between the authors, while 5 different colors seen in this figure represent the collaboration cluster of the authors

Co-authorship in the field of MGBA is shown in Fig. 4, with 5 clusters identified; the size of frame represents the number of publications by an author, and the thickness of lines signifies the extent of collaboration between authors. Of the 6054 authors, 25 had at least ten publications including the most active author Cryan, J.F. with 120 (7.0%) publications.

The 10 most influential journals covering the MGBA research with their IFs are shown in Table 2. The three most influential journals from the top 10 influential journals are *Brain Behavior and Immunity* (49 articles), *Plos One* (34 articles), and *Scientific Reports* (33 articles). Table 3 shows the list of top 20 most-cited articles [56–75] on MGBA. The most prolific institutions were University College Cork (152 articles), McMaster University (67 articles), and INSERM (Institut National de la Santé et de la Recherche Médicale, French National Institute of Health and Medical Research, 43 articles) (Table 4).

**Table 2:** The most productive journals in the microbiome-gut-brain axis research

SCR^a^	Journal	Frequency (%)	IF^b^
1st	*Brain Behavior and Immunity*	49 (2.86)	6.170
2nd	*Scientific Reports*	34 (1.98)	4.011
3rd	*Plos One*	33 (1.93)	2.776
4th	*Gut Microbes*	23 (1.34)	7.823
4th	*World Journal of Gastroenterology*	23 (1.34)	3.411
6th	*Neurogastroenterology and Motility*	22 (1.28)	3.803
7th	*Frontiers in Microbiology*	20 (1.17)	4.259
8th	*Nutrients*	19 (1.11)	4.171
9th	*Advances in Experimental Medicine and Biology*	15 (0.88)	2.126
10th	*Nature Reviews Gastroenterology and Hepatology*	14 (0.82)	23.57

*SCR* Standard competition ranking, *IF* Impact factor

^a^Equal journals have the same ranking number, and then a gap is left in the ranking numbers

^b^Impact factors (IF) based on Journal Citation Reports (JCR) 2018 from Clarivate Analytics

**Table 3:** The 20 most influential articles in the microbiome-gut-brain axis research

SCR^a^	Authors	Title	Year of publication	Source title	Cited by
1st	Nicholson et al. [56]	“Host-gut microbiota metabolic interactions”	2012	*Science*	1490
2nd	Cryan and Dinan [57]	“Mind-altering microorganisms: The impact of the gut microbiota on brain and behavior”	2012	*Nature Reviews Neuroscience*	1204
3rd	Heijtz et al. [58]	“Normal gut microbiota modulates brain development and behavior”	2011	*Proceedings of the National Academy of Sciences of the United States of America*	1116
4th	Hsiao et al. [59]	“Microbiota modulate behavioral and physiological abnormalities associated with neurodevelopmental disorders”	2013	*Cell*	1041
5th	Bravo et al. [60]	“Ingestion of Lactobacillus strain regulates emotional behavior and central GABA receptor expression in a mouse via the vagus nerve”	2011	*Proceedings of the National Academy of Sciences of the United States of America*	1028
6th	Foster and McVey Neufeld [61]	“Gut-brain axis: How the microbiome influences anxiety and depression”	2013	*Trends in Neurosciences*	612
7th	Bercik et al. [62]	“The intestinal microbiota affect central levels of brain-derived neurotropic factor and behavior in mice”	2011	*Gastroenterology*	602
8th	Collins et al. [63]	“The interplay between the intestinal microbiota and the brain”	2012	*Nature Reviews Microbiology*	566
8th	Berer et al. [64]	“Commensal microbiota and myelin autoantigen cooperate to trigger autoimmune demyelination”	2011	*Nature*	566
10th	De Vadder et al. [65]	“Microbiota-generated metabolites promote metabolic benefits via gut-brain neural circuits”	2014	*Cell*	525
11th	Neufeld et al. [66]	“Reduced anxiety-like behavior and central neurochemical change in germ-free mice”	2011	*Neurogastroenterology and Motility*	522
12th	O’Mahony et al. [67]	“Early Life Stress Alters Behavior, Immunity, and Microbiota in Rats: Implications for Irritable Bowel Syndrome and Psychiatric Illnesses”	2009	*Biological Psychiatry*	521
13th	Clarke et al. [68]	“The microbiome-gut-brain axis during early life regulates the hippocampal serotonergic system in a sex-dependent manner”	2013	*Molecular Psychiatry*	476
14th	Sampson et al. [69]	“Gut microbiota regulate motor deficits and neuroinflammation in a model of parkinson’s disease”	2016	*Cell*	455
15th	Tillisch et al. [70]	“Consumption of fermented milk product with probiotic modulates brain activity”	2013	*Gastroenterology*	445
16th	Rhee et al. [71]	“Principles and clinical implications of the brain-gut-enteric microbiota axis”	2009	*Nature Reviews Gastroenterology and Hepatology*	444
17th	Braniste et al. [72]	“The gut microbiota influences blood-brain barrier permeability in mice”	2014	*Science Translational Medicine*	378
18th	Scheperjans et al. [73]	“Gut microbiota are related to Parkinson’s disease and clinical phenotype”	2015	*Movement Disorders*	361
19th	O’Mahony et al. [74]	“Serotonin, tryptophan metabolism and the brain-gut-microbiome axis”	2015	*Behavioural Brain Research*	356
20th	Cryan and O’Mahony [75]	“The microbiome-gut-brain axis: From bowel to behavior”	2011	*Neurogastroenterology and Motility*	347

*SCR* Standard competition ranking

^a^Equal citations have the same ranking number, and then a gap is left in the ranking numbers

**Table 4:** The top ten most productive institutes

SCR^a^	Institute	Country	n (%)
1st	University College Cork	Ireland	152 (8.87)
2nd	McMaster University	Canada	67 (3.91)
3rd	INSERM (Institut national de la santé et de la recherche médicale)	France	43 (2.51)
4th	INRA (Institut National de La Recherche Agronomique)	France	41 (2.39)
5th	University of California, Los Angeles	USA	29 (1.69)
6th	Teagasc - Irish Agriculture and Food Development Authority	Ireland	28 (1.63)
7th	St. Joseph’s Healthcare Hamilton	Canada	26 (1.52)
8th	David Geffen School of Medicine at UCLA	USA	23 (1.34)
9th	The University of North Carolina at Chapel Hill	USA	22 (1.28)
10th	Universite Catholique de Louvain	Belgium	19 (1.11)
10th	University of California, San Diego	USA	19 (1.11)
10th	Københavns Universitet	Denmark	19 (1.11)

^a^Equal institutions have the same ranking number, and then a gap is left in the ranking numbers

## Discussion

This is the first application of bibliometric quantitatively and qualitatively methods regarding the MGBA involving 1713 papers retrieved from Scopus. The results of this bibliometric analysis present a comprehensive overview of the development of the scientific literature in the MGBA field over the past 10 years.

The number of articles concerning MGBA research increased rapidly between 2009 and 2018. This increase is likely related to the many experts in psychiatry, neurology and gastroenterology fields (e.g. Cryan J.F., Dinan T.G., Clarke G., Bienenstock J., Forsythe P., Stanton C., Quigley E.M.M., Bercik P., O’Mahony S.M., Shanahan F., Foster J.A., Moloney R.D., and others) developing their interest in the physiological role of the guts’ microbiota on brain and behavior as an emerging platform for therapeutic intervention of many diseases. Furthermore, the increased number of publications may relate to several hot topics [56–68, 70–72, 74–77] which were published during this period, revealing novel findings that open the door for new areas of investigation. These studies propose novel concepts for treating several conditions such as IBS, autism, depression, multiple sclerosis, auto-immune disease, Parkinson’s disease, and obesity [78–85].

Since 2012, there has been growing research output in the field of MGBA, which is consistent with increasing research activity related to the microbiome in general. Similar findings have been reported in other bibliometric studies [43, 44, 86–89]. A possible underlying explanation for the rising publication numbers is that in 2013 the National Institutes of Health (NIH) launched the second phase of Integrative Human Microbiome Project (iHMP) [90].

Research output related to MGBA most often originated from the United States, as reported in other bibliometric studies regarding microbiome research [43, 44, 86–89]. Our study clearly reveals that the United States is at the forefront of studies on MGBA. The research output from the USA may be associated with the wide range of researchers with an interest within this field and a substantial amount of financial support to researchers. In 2013 the USA launched a special research project on gut microbiota-brain axis [91]. Since then, there has been increasing neuroscience interest in the role of gut microbiota on animal and human brain behavior and cognitive development [92, 93]. Ireland featured as the second most prolific nation and this might be related to Professor John F Cryan and Professor Ted Dinan, with their team who are the most active authors in this field, and principal investigators at the Alimentary Pharmabiotic Centre (APC) in University College Cork. [94] The APC is funded by Science Foundation Ireland (SFI) [75], and has conducted studies in collaboration with several companies including GlaxoSmithKline, Cremo, Suntory, Pfizer, Wyeth and Mead Johnson which consequently provided more funding for conducting research in the field of psychobiotics [75], thus may contribute to increasing number of publications regarding gut microbiota-brain axis.

The number of citations for the top 20 articles in the current study varied from 1490 to 347, which is higher range of citations than in other medical fields such as mobile-health [95], toxicology [28], social media in psychology [96], parasitic diseases [51, 97], and viral diseases [98–100]. Additionally, it also reveals that researchers paid great attention on the MGBA mostly in recent years, and published several outstanding articles on top-ranking journals in the medical field such as Science [56] and Nature [64]. The most cited article is “Host-gut microbiota metabolic interactions” a review by Nicholson et al., 2012 [56], published in *Science*, where the authors suggest that the manipulation of the gut microbiota to optimize new therapeutic strategies could control many diseases and improve health. The second most cited article “Mind-altering microorganisms: The impact of the gut microbiota on brain and behavior” was published in the *Nature Reviews Neuroscience* in 2012 by Cryan and Dinan [57], where the authors suggest that the concept of a microbiota-gut-brain axis may lead to the development of novel therapeutics for management of several neurological and psychiatric disorders.

Finally, there are some limitations for our study findings. First, the search was limited to publications listed in Scopus, which is the largest biomedical database and the most frequently used database for bibliometric analyses, although it might not contain all publications relevant to MGBA research. MGBA publications that do not include this term or its synonyms in the title, abstract or key words might not be taken into account for our analysis. Secondly, a general limitation of the bibliometric approach is that there is no weighting to take account of the quality or scientific rigor of any individual publication. Despite these limitations, we still consider that the findings offer a valid representation of MGBA research output at a global level.

## Conclusions

The characteristics of the MGBA related publications from 2009 to 2018 are investigated through the bibliometrics analysis based on the Scopus database. This study demonstrates that the research on the MGBA has been becoming progressively more extensive at global level over the past 10 years. Overall, our study found a large amount of work on MGBA, focused on immunomodulation, irritable bowel syndrome, and neurodevelopmental disorders. Despite considerable progress illustrating the communication between the gut microbiome and the brain over the past 10 years, many issues remain to fully realize their relevance for therapeutic intervention of many diseases.

## Data Availability

Not applicable.

## Abbreviations

IBS: Irritable bowel syndrome
IFs: Impact factors
JCR: Journal citation reports
SCR: Standard competition ranking
SPSS: Statistical package for social sciences
